# Finite-Aperture Limits for Yaw Estimation in Confocal Non-Line-of-Sight Imaging

**DOI:** 10.3390/jimaging12060248

**Published:** 2026-06-02

**Authors:** Riccardo Romanelli, Lorenzo Francesco Livi, Francesco V. Pepe, Giacomo Sorelli, Enea Mauri, Milena D’Angelo, Massimiliano Proietti

**Affiliations:** 1 Dipartimento Interuniversitario di Fisica, Università degli Studi di Bari Aldo Moro, 70125 Bari, Italy; 2 Leonardo Innovation Hub, Quantum Technologies Lab, Leonardo S.p.A., 00131 Rome, Italy; 3 Leonardo Electronics Division, Leonardo S.p.A, 50013 Campi Bisenzio, Italy; 4 Istituto Nazionale di Fisica Nucleare, Sez. di Bari, 70125 Bari, Italy; 5 Fraunhofer IOSB, Ettlingen, Fraunhofer Institute of Optronics, System Technologies and Image Exploitation, Gutleuthausstr. 1, 76275 Ettlingen, Germany; giacomo.sorelli@iosb.fraunhofer.de

**Keywords:** non-line-of-sight imaging, time-of-flight, confocal transient imaging, finite wall aperture, yaw estimation, fisher information, cramér–rao bound, forward model

## Abstract

Non-line-of-sight (NLOS) time-of-flight imaging can recover hidden-scene geometry from the transient image measured on a relay wall. While the finiteness of the relay wall is known to constrain reconstruction, its impact on the angular estimation of the target has not yet been characterized. We address this gap through two complementary analyses. First, we derive a simple geometric visibility criterion based on a vertical switch line on the wall, and identify the angular range over which the finite wall still preserves the main transient features needed for a unique planar reconstruction. Second, we quantify angular sensitivity through the Fisher information of the normalized transient shape, showing that yaw sensitivity is not distributed uniformly across the wall and decreases smoothly as the most informative part of the measurement is progressively clipped by the finite aperture. In this way, the switch-line threshold emerges as a geometric transition rather than a complete loss of angular information. Our findings help clarify the limits of angular estimation with finite relay walls and provide guidance for interpreting and designing confocal NLOS measurements.

## 1. Introduction

Modern imaging systems go beyond the standard acquisition of the bi-dimensional intensity distribution by encoding richer information about the propagating light. For example, light-field imaging samples both spatial information and propagation direction, enabling computational refocusing, viewpoint changes, and advanced 3D imaging capabilities [1,2,3,4]. More recently, correlation-based classical and quantum extensions of light-field imaging have enabled improved resolution and volumetric imaging capabilities [5,6,7,8,9]. These approaches extend conventional cameras yet remain confined to line-of-sight observations: scene geometry hidden behind an occluder remains inaccessible.

Non-line-of-sight (NLOS) imaging uses an umbrella of approaches to recover hidden 3D scenes from multiply scattered light collected on a diffusive relay wall [10,11,12]. In this context, coherent and passive settings exploit speckle-based methods together with passive schemes based on spatial coherence or ambient illumination [13,14,15,16,17]. On the other hand, the more common active modality, which is the focus of this paper, relies on a pulsed source to illuminate the wall and on an ultrafast time-of-flight detector to record the returning transient image, i.e., the time-resolved response of the hidden scene to the illumination pulse [18]. By inverting these time-stamped light echoes, the hidden scene can be reconstructed, effectively turning the rough wall into a virtual imaging surface [19,20,21,22,23,24,25].

Scattering walls thus open access beyond occluders, but at the cost of angular information. Similarly to flood-illumination transient imaging [26], each camera viewpoint on the sampled wall acts as a bucket detector: its transient histogram collects delayed and scaled contributions from the whole hidden scene. A single transient does not correspond to a single point in space, but to an integral over points with equal round-trip delay, leading to a severely ill-posed inverse problem. In practice, the ambiguity is reduced by sampling many wall positions, either sequentially or in parallel [20,22,27,28,29,30], and by using reconstruction methods that exploit extra structure in the measurements or in the scene, such as occlusion information, regularization, or learned priors [31,32,33,34,35,36,37,38].

Even then, a finite wall aperture and sampling grid necessarily capture only a subset of light paths. This leads to the missing-cone problem [19,23,27,39] and, for planar targets, makes the limitation strongly yaw-dependent, with reconstruction possible only when the plane normal intersects the sampled wall. While this picture provides a useful visibility bound, the practical severity of the limit still depends on the reconstruction method and on the information carried by the measurement.

Additional directional cues, such as polarization, and alternative formulations based on transient discontinuities have been shown to improve recovery beyond standard volumetric inversion [40,41,42,43,44,45]. This motivates studying not only whether reconstruction is possible, but also which geometric information remains in the transient when a finite wall clips the measurement.

**Figure 1:**
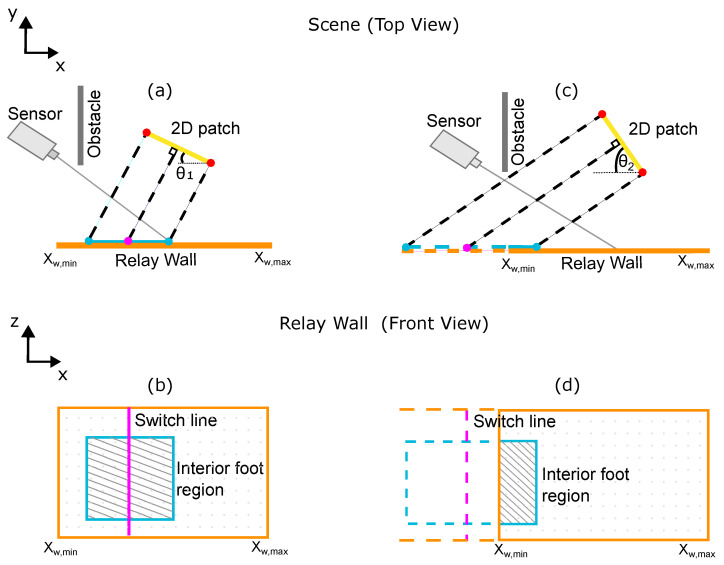
Geometry of the finite-wall yaw problem and of the two wall features used in the analysis. (**a**,**c**) Top view in the x − y plane for two yaw angles. The orange segment is the sampled relay wall, the magenta point is the switch-line location, and the cyan segment marks the interior-foot region, namely the wall subset whose orthogonal projection onto the plane falls inside the finite patch. In (**a**), both features lie inside the sampled wall. In (**c**), increasing yaw moves them toward the wall boundary, and part of this structure leaves the observed aperture. (**b**,**d**) Corresponding front views on the relay wall. The magenta vertical line is the switch line, while the cyan region marks the interior-foot region, with the solid portion denoting the part still sampled by the wall and the dashed portion the part lost outside the aperture. The finite wall aperture is shown in orange. The switch line provides the geometric limit for preserving the internal support-bound swap needed for unique scene reconstruction on the finite wall. However, angular information does not disappear at that threshold: it can still remain in the measured transient beyond the switch-line limit, mainly through the wall portion that still overlaps the interior-foot region.

In this work, we address this question in the time domain using a closed-form transient model for a finite isotropic planar patch observed on a finite confocal relay wall. In this setting, each wall bin acts as both transmitter and receiver, and the model admits closed-form analysis for the simple target considered [46], unlike more general non-confocal geometries [47,48]. At a single wall bin, the transient support is bounded by the first and last patch points that can contribute, which may be either interior points of the patch or endpoints (patch edges). These support limits carry useful geometric cues, but they are not unique by themselves. When many wall bins are observed together, the wall records how these support bounds move across the aperture, and this reveals a much richer structure of the target. We address this issue with two complementary approaches. The first one is illustrated in Figure 1. For yaw rotations, the wall transient image is structured around a vertical switch line, namely the wall position where signals from the target endpoints exchange their order of arrival in the measured transient image. As long as this line stays inside the sampled wall, the measured transient is a clipped part of the ideal one, but still captures the main support structure of the finite plane. If it moves out of the wall, the transient may still carry information about the target, but it is no longer sufficient for a unique planar reconstruction on the sampled aperture when using general reconstruction algorithms that do not impose strong a priori assumptions on the target shape. This provides a time-domain geometric counterpart to the visibility limit discussed in the literature [39], while also clarifying the finite-wall ambiguity that appears beyond it.

The second approach complements this geometric criterion with a continuous, information-based analysis based on shape-only Fisher information. When yaw is treated as the unknown parameter of the globally normalized transient, the information is not distributed uniformly across the wall, but is concentrated mainly inside the interior-foot region, where the first contribution comes from the patch interior rather than from an endpoint. This is captured by the Fisher information associated with the wall, which decreases smoothly as yaw increases; in fact, the most informative wall region is progressively clipped by the finite aperture, while the switch-line exit marks a later geometric transition rather than a complete loss of information.

## 2. Materials and Methods

### 2.1. Geometric Criterion from Transient Support and Switch-Line Visibility

#### 2.1.1. Target and Wall Geometry

We consider a vertical planar patch in front of a relay wall, and we study how well its yaw can be inferred from time-resolved confocal NLOS measurements.

Figure 1 summarizes the geometry and the notation used in this section. The figure also introduces the two wall features used throughout the analysis: the switch line, which marks where the endpoint ordering changes, and the interior foot region, which identifies the wall subset whose orthogonal projection onto the plane falls inside the finite patch.

The target lies in $R^{3,+}$ (half-space y>0), is laterally centered at $x_{c}$, placed at stand-off distance d>0 from the wall plane y=0, translated along *z* by $z_{0}$, and yawed by an angle θ about $\hat{z}$. The patch has width *ℓ* (along the lateral direction explored during yaw) and height *h* (along $+\hat{z}$). The relay wall is the sampled portion of the wall used for the NLOS measurement. It is observed over the finite rectangular window

(1) $$\left(x_{w},z_{w}\right)\in \left[x_{w,\min},x_{w,\max}\right]\times \left[z_{w,\min},z_{w,\max}\right],$$

discretized into wall bins, where each wall bin $w=(x_{w},0,z_{w})$ is one sampled wall location and acts as both illumination point and detection point in the confocal acquisition.

The main limitation studied here is the finite wall aperture. As yaw changes, the most informative features of the transient image move across the wall. If they remain inside the sampled wall, the measurement still preserves the geometric cues needed to localize the plane. If they move outside, the measured transient becomes a cropped version of the ideal one, and the reconstruction becomes more ambiguous.

#### 2.1.2. Transient Support and Switch-Line Criterion

For a fixed wall bin w, the transient is nonzero only between the first and the last patch points that can contribute to that bin. Its temporal support is therefore

(2) $$t\in \left[\frac{2}{c}\min_{p\in P(\theta )}∥w-p∥,\;\frac{2}{c}\max_{p\in P(\theta )}∥w-p∥\right],$$

where P(θ) denotes the set of patch points that contribute under the adopted confocal model. The lower bound is set by the closest contributing point to the wall bin. If the orthogonal projection of w onto the target plane falls inside the finite patch, this first point lies in the interior of the patch. Otherwise it is clamped to one of its edges. The upper bound is simpler: for a finite planar patch, the farthest contributing point is always one of the corners. Therefore, the corners limit the transient support for every wall bin and for every yaw angle. In this sense, the endpoint events provide the wall with the clearest geometric signature of the finite target extent.

At one wall bin, these support bounds carry useful geometric information, but they are not unique by themselves. Different planar configurations may produce similar first and last delays, or a similar support width. The ambiguity is reduced only when many wall bins are observed together, because the wall then records how these support bounds move across the aperture. A detailed derivation of the closed form transient model, both for one wall bin and for the full wall, together with a geometric interpretation of the transient structure, is provided in the Supplementary Materials.

For yaw rotations, the most important changes come from corners at the same height. Let $p_{-}\left(\theta \right)$ and $p_{+}\left(\theta \right)$ be the left and right endpoints of the bottom edge,

(3) $$p_{\pm }\left(\theta \right)=\left(x_{c}\pm \frac{\ell }{2}\cos\theta ,\;d\pm \frac{\ell }{2}\sin\theta ,\;z_{0}\right).$$

As the wall bin moves, the distances to the corners change, and at one wall location they become equal. There, the corner that sets the transient boundary switches. Since the target rotates about the vertical $\hat{z}$ axis, the equal-range condition for same-height endpoint pairs is independent of $z_{w}$. In the present model, yaw is defined as a rotation of the finite patch about its center, whose lateral coordinate is $x_{c}$. With this convention, the normal to the target plane passing through the patch center intersects the relay wall at

(4) $$x^{★}\left(\theta \right)=x_{c}+d\tan\theta .$$

This is the switch line of the transient: a vertical line on the wall. Under the same center-rotation convention, the same expression holds for both the bottom and top endpoint pairs, so the whole envelope inversion is controlled by a single wall line.

This line also has a direct geometric meaning: it is the wall line reached by the normal to the target plane passing through the center of the patch. In this sense, the switch-line criterion is fully consistent with the visibility picture of Liu et al. [39]. When this line is sampled by the wall, the measurement still contains the main symmetry point of the planar transient. When it is not sampled anymore, that structure is lost from the acquired aperture.

An internal switch is present only if this line falls inside the sampled wall span,

(5) $$x_{w,\min}\leq x^{★}\left(\theta \right)\leq x_{w,\max}.$$

For θ∈[0,π/2], this gives the yaw window

(6) $$\theta \in \left[\arctan\;\left(\frac{x_{w,\min}-x_{c}}{d}\right),\;\arctan\;\left(\frac{x_{w,\max}-x_{c}}{d}\right)\right].$$

Outside this window, the target may still return light, but the sampled wall no longer contains the change in endpoint ordering. Beyond this limit, the data may still constrain the target under additional priors, but the sampled aperture no longer contains the full support transition required for an unambiguous planar interpretation.

#### 2.1.3. Loss of Support-Bound Information on a Finite Wall

When the switch line lies inside the sampled wall, the wall sees the change in endpoint ordering across its bins. This makes the transient support vary across $x_{w}$ in a way that still reflects the geometry of the planar patch. In this regime, the measured transient keeps the main geometric feature needed for a unique planar interpretation.

When the switch line moves outside the sampled wall, this change is no longer observed. The wall records only a cropped portion of the ideal transient support. The support observed at the two wall edges then plays the role of an effective boundary for the measured data, even though it is not generated by a real corner of the patch.

The result is a finite ambiguity region: several nearby planes can produce the same measured transient, even though they would be distinguishable with a wider wall. The switch-line condition is therefore a binary criterion for unique planar reconstruction on a finite wall. Beyond it, the transient still carries information on the target, but not enough for a unique planar reconstruction on the sampled aperture.

#### 2.1.4. Closed-Form Transient Model Used for the Simulations

The simulated transient images are computed from a closed-form confocal model. For one wall bin, the finite patch response is assembled as a sum of rectangular block contributions,

(7) $$\tau \left(t;\theta ,\ell ,h,d,x_{o},z_{o}\right)=\sum_{i=1}^{2}\sum_{j=1}^{2}f\left(t;U_{i},V_{j},\lambda \right).$$

The full wall transient is then obtained by evaluating the same expression over the sampled relay wall and by convolving it with the temporal response of the system,

(8) $$V\left(t,z_{w},x_{w};\theta \right)=\left[\tau (\cdot ;\theta ,\ell ,h,d,x_{0}-x_{w},z_{0}-z_{w})*h\right]\left(t\right).$$

The derivation of the kernel *f*, the block intervals $U_{i},V_{j}$, and the distance λ is given in the Supplementary Material. All numerical simulations, Fisher-information calculations, data analysis, and figure generation were performed using Python 3.10.14.

### 2.2. Fisher Analysis of Yaw Sensitivity

The geometric criterion above marks the loss of unique planar reconstruction on a finite wall, but not necessarily the complete loss of yaw information. Beyond the switch-line threshold, the transient may still retain a weaker dependence on angle. This motivates introducing a continuous visibility measure. To describe how yaw sensitivity degrades more gradually, we complement it with a Fisher information (FI) analysis. We study yaw from the shape of the transient, not from its total energy. Let $V_{n,W}\left(\theta \right)\geq 0$ be the transient sampled at time bin *n* and wall bin *W*. We first normalize it over all time bins and all wall bins

(9) $${\tilde{V}}_{n,W}\left(\theta \right)=\frac{V_{n,W}\left(\theta \right)}{\sum_{W}\sum_{n}V_{n,W}\left(\theta \right)}\;\;\;\mathrm{with}\;\;\;\sum_{W}\sum_{n}{\tilde{V}}_{n,W}\left(\theta \right)=1.$$

This global normalization removes the overall photon budget and keeps only how the signal is distributed over time and wall position. For a single wall bin, we can also define its local FI

(10) $$I_{W}\left(\theta \right)=\sum_{n}V_{n,W}\left(\theta \right){\left[\partial_{\theta }\log V_{n,W}\left(\theta \right)\right]}^{2}.$$

It is a useful quantity to identify which bins are locally informative, but it does not describe the full wall response. The main quantity used is the wall FI

(11) $$I\left(\theta \right)=\sum_{W}I_{W}\left(\theta \right)=\sum_{W}\sum_{n}{\tilde{V}}_{n,W}\left(\theta \right)\;{\left(\partial_{\theta }\log{\tilde{V}}_{n,W}\left(\theta \right)\right)}^{2}.$$

This quantity measures the total yaw sensitivity of the full transient image and therefore accounts for the information collected over the entire sampled wall. In practice, the derivative $\partial_{\theta }$ is evaluated numerically on the sampled yaw grid used in the simulations.

For readability, we also report the corresponding per-photon uncertainty proxy in degrees,

(12) $$\sigma_{\theta }\left(\theta \right)=\frac{180/\pi }{\sqrt{I(\theta )+\varepsilon }},$$

where ε>0 prevents numerical divergence when the FI becomes very small. The factor 180/π converts the angular uncertainty from radians to degrees. Smaller $\sigma_{\theta }$ corresponds to higher yaw sensitivity.

## 3. Results

### 3.1. Finite-Wall Ambiguity and Reconstruction Behaviour

To show how the switch line limit appears in practice, we reconstruct the same simulated transient data with two standard NLOS methods. The first is f-k reconstruction (FK), a Fourier-domain inversion method that reconstructs the hidden volume through a wave-based migration step. The second is backprojection (BP), which reconstructs the scene by summing the measured transient contributions along the corresponding constant-delay surfaces [20,27]. We use them here only as representative inversion schemes, in order to see how the same finite-wall loss of structure appears in two different reconstructions.

**Figure 2:**
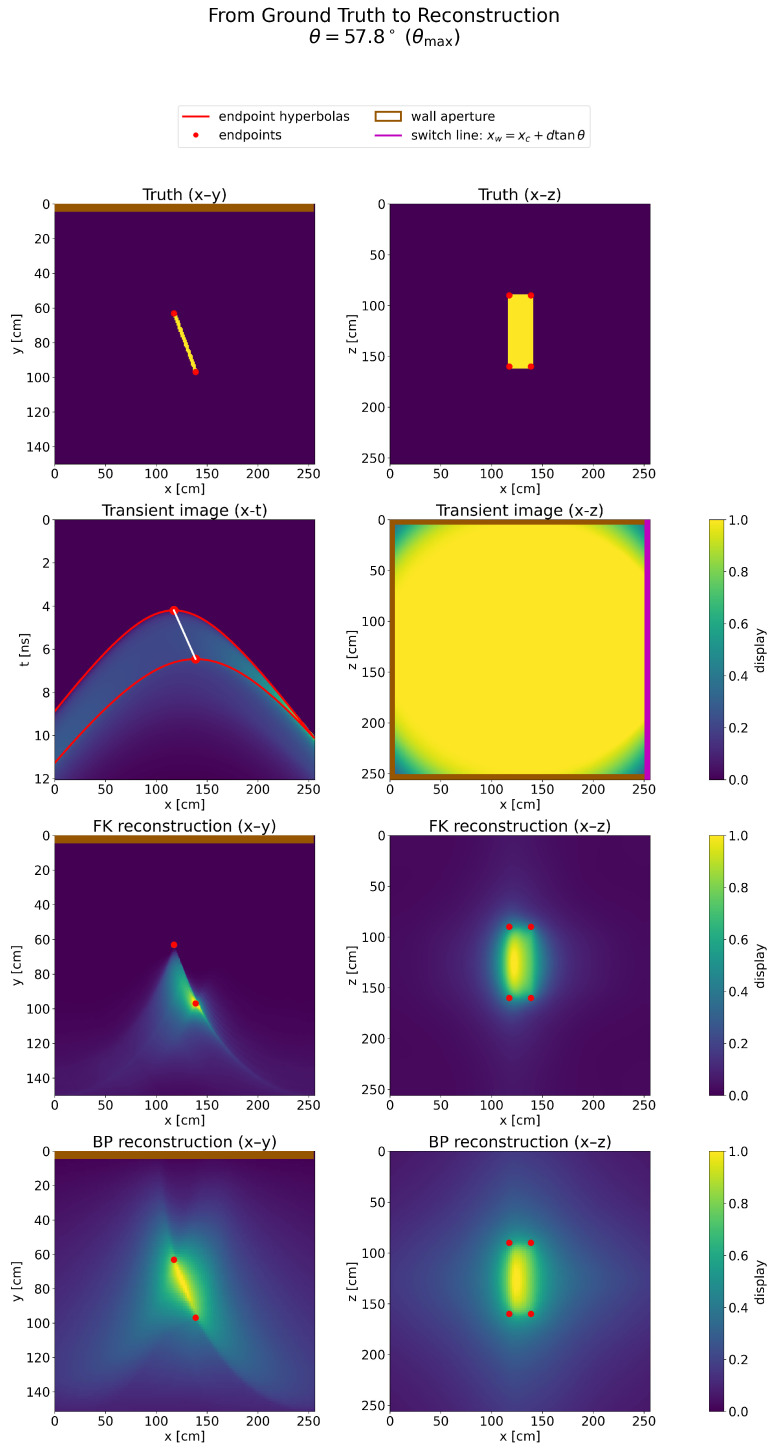
Boundary case at $\theta =57.8^{\circ }$, equal to the geometric switch-line limit for the adopted wall span. The top row shows the ground-truth patch in the x − y, x − z, and 3D views. The second row reports the transient projections XT and ZX, together with the geometric overlays: the red hyperbolas, corresponding to the traces of the target endpoints in the transient image, the wall aperture in orange, and the switch line in magenta. Here the switch line $x^{★}\left(\theta \right)=x_{c}+d\tan\theta$ reaches the right edge of the sampled wall, so the endpoint swap is still present but only at the boundary of the aperture. The third and fourth rows show the corresponding f-k and backprojection reconstructions, which remain localized but already indicate a more fragile reconstruction regime than at smaller yaw.

For the simulated geometry $d=80\;\mathrm{cm},\;x_{c}=128\;\mathrm{cm},\;\left[x_{w,\min},x_{w,\max}\right]=\left[0,255\right]\;\mathrm{cm}$, From Equation (4), the last yaw for which the switch line still lies on the sampled wall is $\theta_{\max}\approx 57.8^{\circ }.$ Figure 2 shows this boundary case. The magenta switch line reaches the right wall edge. The red hyperbolas, corresponding to the traces of the target endpoints in the transient image, still meet, but only at the boundary of the sampled aperture. The transient remains compact, and both the reconstructions still recover a localized scene. However, the reconstruction is already more fragile than at smaller yaw, because this is the last configuration for which the sampled wall still contains the endpoint swap.

**Figure 3:**
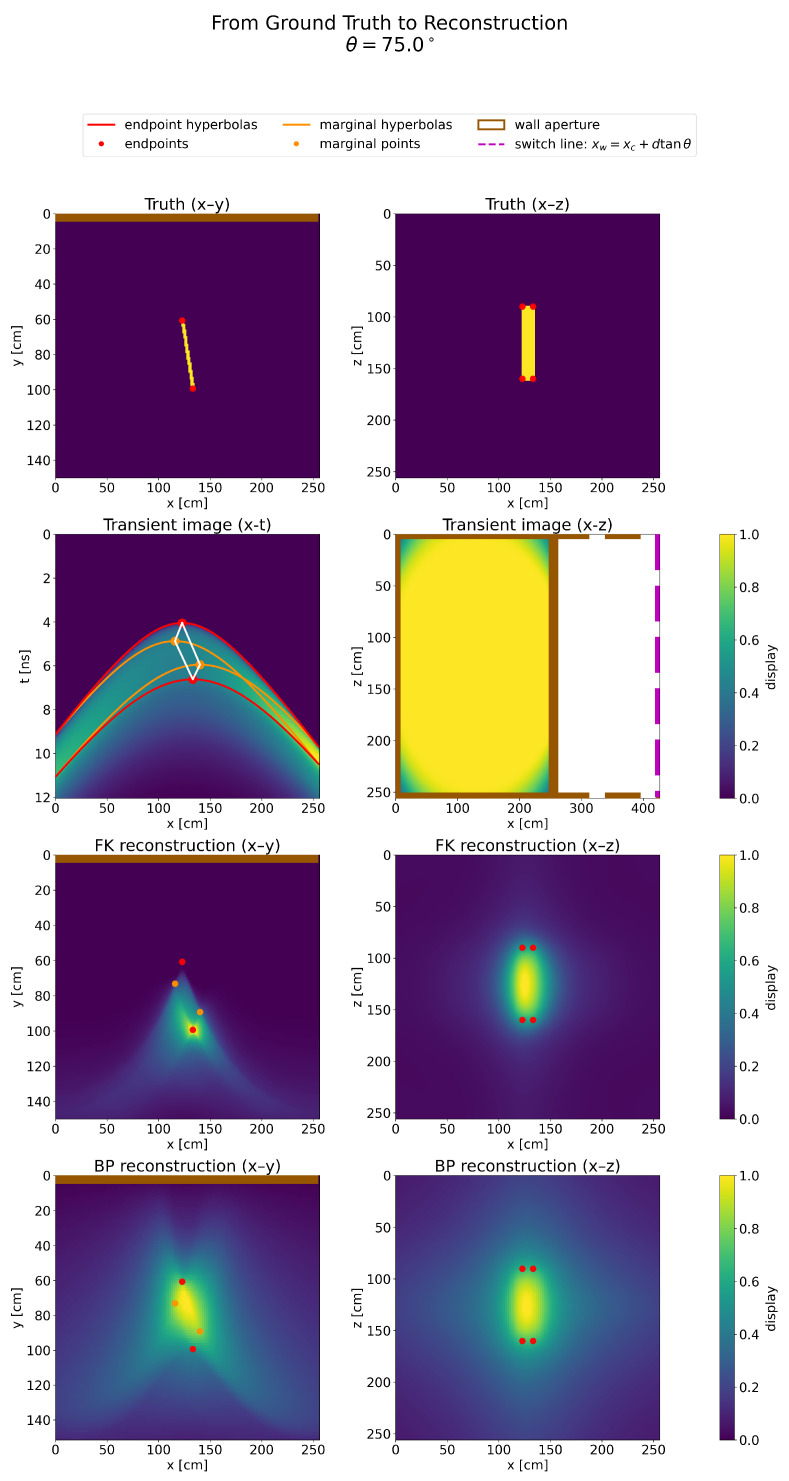
Case beyond the geometric visibility limit at $\theta =75^{\circ }$. The transient panels show that the switch line has moved outside the sampled wall, so the endpoint ordering no longer changes inside the acquired aperture. The wall therefore records only a cropped part of the ideal transient support. The white regions in the transient images correspond to wall positions outside the sampled aperture and therefore to data that are not acquired. The red hyperbolas are the traces of the target endpoints in the transient image. The orange marginal curves and points mark the support induced by finite-aperture clipping. In this regime the scene is no longer recovered as a unique plane: f-k reconstruction retains mainly an edge-like remnant, whereas backprojection yields a finite ambiguity region compatible with the measured data.

Figure 3 shows what happens beyond this limit. At $\theta =75^{\circ }$, the switch line lies outside the sampled wall, so the wall records only a cropped part of the ideal transient support. In the transient images, the white regions indicate wall positions outside the sampled aperture and therefore correspond to data that are not acquired.

Here, the wall-limited curves become important. The two edges of the wall select opposite branches of the cropped endpoint hyperbolas and connect them through virtual boundary events. In the transient image, these appear as marginal hyperbolas and marginal points, which define a finite region of support that is still compatible with the data, even though the unique endpoint exchange has disappeared.

Reconstruction methods reveal the same finite-wall limitation in different ways. FK reconstruction appears as a weak edge-like remnant. This is consistent with the Fourier-domain nature of the method: a finite wall aperture and sampling grid produce a missing cone of unmeasured spatio-temporal frequencies [39], so only the part of the hidden-scene spectrum that lies inside the measured subspace can be reconstructed [19,23,27,39]. Since the patch spectrum is a line through the Fourier origin that rotates with the patch itself, much of this line falls outside the local measurement cone at large yaw.

In BP reconstruction, the same loss of uniqueness appears instead as a broader nonzero volume. The data therefore remain informative, although in a weaker and more ambiguous way. The target has not disappeared from the data, but it is no longer recovered as a unique plane.

These results clarify the main limitation of the switch line criterion. It tells us when the clearest support feature is lost, but it does not tell us how much yaw information still remains after that point. For this reason, the next section introduces FI as a continuous measure of yaw sensitivity.

### 3.2. Experimental Validation of the Transient Model

To validate the forward model underlying the geometric criterion, we compare experimental NLOS measurements with the corresponding simulated confocal transients and reconstructions. The measured transient structures and the reconstruction behaviour follow the transition predicted by the model.

**Figure 4:**
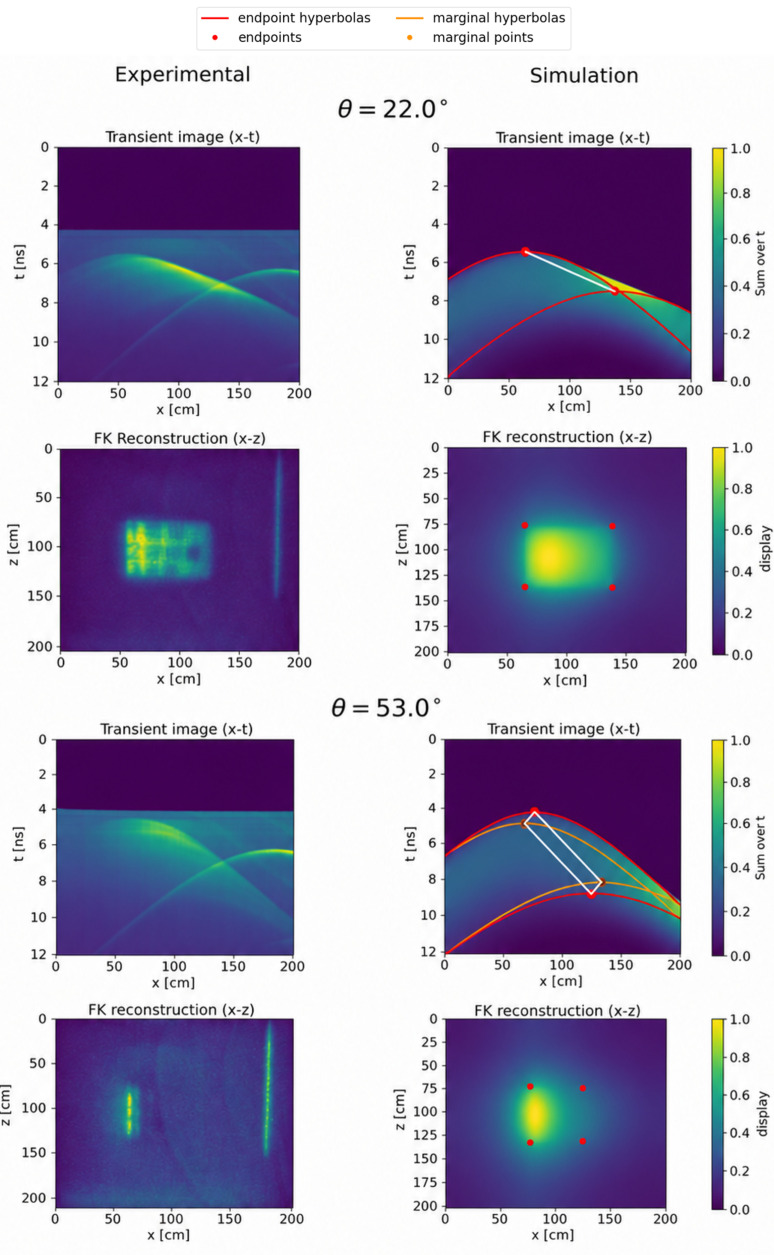
Experimental and simulated validation of the confocal transition model on a black-on-white USAF-style chart target. The left column shows experimental measurements and f-k reconstructions, while the right column shows the corresponding simulated transients and reconstructions. Two yaw angles are shown: $\theta =22^{\circ }$ and $\theta =53^{\circ }$. In the simulated transient images in the right column, red marks denote endpoint traces and points, while orange marks denote marginal traces and points. For the 2 m×2 m relay wall and d=1 m, the geometric switch-line criterion predicts a reconstruction limit of $\theta_{\max}=45^{\circ }$. At $\theta =22^{\circ }$, both experiment and simulation support a stable reconstruction of the target structure. At $\theta =53^{\circ }$, which lies beyond this limit, the reconstruction is strongly degraded, consistently with the finite-aperture transition predicted by the model.

Figure 4 shows two representative yaw angles for a black-on-white USAF-style chart target. At $\theta =22^{\circ }$, the target is reconstructed with the expected planar structure. At $\theta =53^{\circ }$, which is beyond the reconstruction limit $\theta_{\max}=45^{\circ }$ predicted by the geometric switch-line criterion for the 2 m×2 m wall and d=1 m, the reconstruction is strongly degraded. The main experimental parameters and components are listed in Table 1 and Table 2, respectively.

### 3.3. Fisher Information as a Continuous Measure of Yaw Observability

The numerical settings used in this analysis are summarized in Table 3.

For this reference scene, we compute the shape FI of the globally normalized transient introduced in Section 2.2. The expected measured transient is $\mu_{i}\left(\theta \right)=S_{i}\left(\theta \right)+\beta$, where $S_{i}\left(\theta \right)$ is the simulated signal and β is a constant Poissonian background floor. The normalized transient and the corresponding shape FI are

(13) $$I_{\mathrm{shape}}\left(\theta \right)=\sum_{i}\frac{1}{p_{i}\left(\theta \right)}{\left[\frac{\partial p_{i}\left(\theta \right)}{\partial \theta }\right]}^{2},\;\;\;\;\;\;p_{i}\left(\theta \right)=\frac{\mu_{i}\left(\theta \right)}{\sum_{j}\mu_{j}\left(\theta \right)}.$$

The raw Poisson FI contains a photon count term and a shape term. Under the isotropic target model considered here, the angular dependence is dominated by the shape term, while the photon budget mainly acts as a scale factor. Consequently, once the curves are normalized for display, the shape FI and the raw Poisson FI nearly overlap.

#### 3.3.1. Extracting Information from the Wall

We now examine how yaw information is distributed over the sampled relay wall. Each wall bin, namely each sampled wall location in the confocal grid, provides a local transient trace and therefore a local contribution to yaw sensitivity.

**Figure 5:**
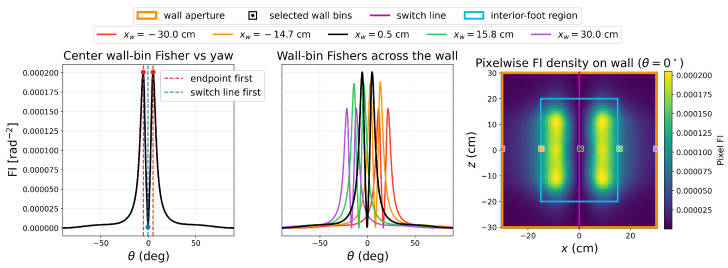
Local yaw Fisher information structure from the one-bin analysis. (**Left**): Fisher information of the wall bin closest to the wall center versus yaw. The dashed red lines mark the angles where the first contributing point reaches a patch endpoint, and the dashed blue line marks the central minimum where the first contribution lies in the patch interior. (**Middle**): same analysis for five wall bins on the same wall row. Symmetric wall positions give mirrored responses, while each bin reaches its maximum at a different yaw. (**Right**): pixelwise Fisher information density on the wall at $\theta =0^{\circ }$. The orange rectangle marks the sampled wall, the magenta line marks the switch line, the dashed cyan box marks the interior-foot region, and the highlighted points, labelled by a black square with a central dot, are the five bins used in the middle panel. The information is concentrated in two interior bands, while bins near the switch line and bins outside the interior-foot region are less informative.

Figure 5 shows why a single wall bin is only a local observability probe.

The left panel reports the FI curve of the wall bin closest to the wall center. The curve has two symmetric maxima and a sharp minimum at the center. The two maxima occur when the earliest contributing point comes from a patch endpoint. At those angles the local transient changes most rapidly with yaw, so the FI becomes large. By contrast, the minimum occurs when the first contributing point lies in the patch interior. In that configuration the local trace is most symmetric, and the first-order yaw sensitivity is strongly reduced.

In the middle panel, we extend the same analysis to five wall bins spread across the sampled wall and highlighted in the red panel. The curves are not aligned. Each wall position has its own most informative angle because each bin sees a different local transient support and a different motion of the first contributing point over the finite patch. Bins placed symmetrically with respect to the switch line produce mirrored FI curves, which reflects the symmetry of both the geometry and the transient. The center bin is the least informative one around $\theta =0^{\circ }$, because the two sides of the target contribute most symmetrically there, so small yaw changes produce only a weak change in the local transient.

The right panel shows the pixelwise FI density on the relay wall at $\theta =0^{\circ }$. The information is clearly not distributed uniformly. Most of it is concentrated in two vertical bands inside the interior-foot region, that is, the wall region where the orthogonal foot of the wall bin onto the plane falls inside the finite patch. These bands lie away from the switch line and correspond to wall bins whose local transient changes most strongly when yaw shifts the first contribution. The bins near the switch line carry much less information. Their local signals receive more balanced and competing contributions from the two sides of the patch, so the local shape changes more weakly with angle. Outside the interior-foot region the FI density also drops, because the local transient becomes more cropped and increasingly edge-dominated. The whole pattern is symmetric about the switch line, as expected for the untilted reference pose.

Overall, the figure shows that one-bin FI is a local quantity. A wall bin can be very informative, but only over a limited angular range and only at specific wall locations. The informative part of the sampled wall is structured, not uniform.

#### 3.3.2. Wall Fisher Information as a Continuous Visibility Indicator

**Figure 6:**
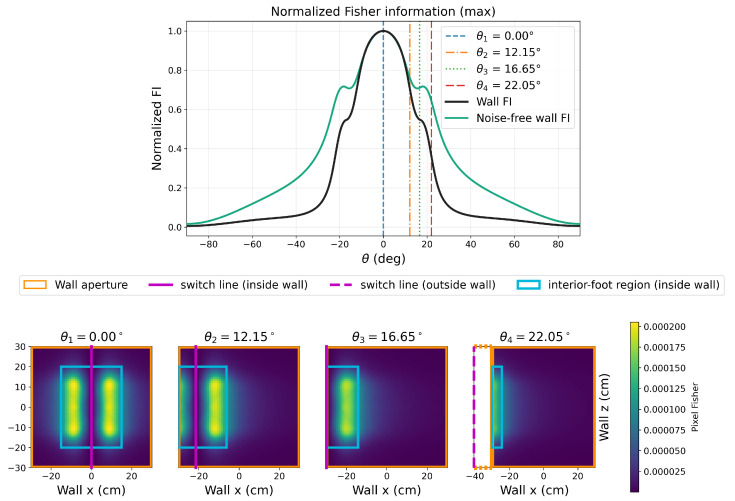
Wall-level FI as a continuous visibility indicator. Top: normalized wall FI versus yaw for the observed transient with background and for the corresponding noise-free reference. The background reduces the measurable FI, while the overall angular trend remains governed by finite-aperture clipping. The vertical dashed lines mark four representative angles used in the wall maps below. Bottom: pixelwise FI density on the wall for those four yaw values. The orange rectangle shows the wall aperture, the magenta line is the switch line, and the dashed cyan box marks the interior-foot region visible on the wall. At $\theta_{1}=0^{\circ }$, two symmetric informative bands are present. At $\theta_{2}$, one band has already been excluded by the finite wall, causing the steepest FI decrease. At $\theta_{3}$, the switch line reaches the wall boundary, but one informative region still survives, so the information decreases more slowly. At $\theta_{4}$, the switch line is outside the wall, yet the wall FI remains nonzero because the remaining cropped transient still carries yaw sensitivity.

Figure 6 shows how the FI of the globally normalized transient changes with yaw when computed over the full sampled wall. Unlike the one-bin curves, it provides a single measure of how much yaw information remains in the full transient. The top panel reports the normalized wall FI versus yaw, comparing the observed case with background and the corresponding noise-free reference. Both curves show the same finite-aperture trend: the FI is largest near $\theta =0^{\circ }$, where the two informative wall bands are both visible inside the sampled wall, and decreases as yaw increases. The background reduces the measurable FI, as expected, but does not change the main angular behaviour, which is governed by the progressive loss of the most informative wall regions.

The bottom row helps interpret this trend in the wall domain. At $\theta_{1}=0^{\circ }$, the FI density shows two symmetric informative bands. At $\theta_{2}$, one band is already partly lost because the corresponding region has moved outside the wall. Around $\theta_{3}$, the switch line reaches the wall boundary, but one informative region is still visible. At $\theta_{4}$, the switch line is outside the wall, yet the wall FI remains nonzero because the cropped transient still changes with yaw. The FI approaches zero only as $\theta \to 90^{\circ }$. This comparison shows why the geometric and FI criteria are complementary. The switch-line condition tells us when the sampled wall no longer contains the internal change in endpoint ordering. The FI measures instead how strongly the observed transient still responds to yaw. In the present example, yaw sensitivity decreases before the switch line exits the wall and remains nonzero after that point.

## 4. Discussion and Conclusions

In this work, we introduced two complementary approaches to study yaw limits caused by a finite relay-wall aperture. The first one is a geometric criterion based on the switch line, namely the wall location where the endpoint ordering of the transient support changes across wall bins. The second one is a Fisher information analysis of the globally normalized transient, which quantifies continuously the change of yaw sensitivity as the transient is progressively clipped by the wall.

Together, these results support the following picture. The finite sampled relay wall limits yaw observability not only by reducing the collected signal, but also by removing the wall regions where the transient changes most clearly with angle. In the geometric analysis, this loss is marked by the switch line leaving the sampled wall. The corresponding yaw range depends directly on the wall span, the target stand-off distance, and the lateral target position. The Fisher analysis adds to this result an important insight: the switch-line threshold marks the loss of the clearest internal support change, but it does not coincide with a complete loss of yaw information. In the examples studied here, yaw sensitivity starts to weaken before that threshold and remains nonzero after it. This means that loss of geometric uniqueness and loss of useful yaw information do not occur at exactly the same angle.

The simulations also show that the remaining information appears differently depending on the reconstruction method. Beyond the switch-line limit, f-k reconstruction mainly preserves edge-like components, while back-projection yields a broader nonzero volume of compatible hypotheses. Both analyses reflect the same finite-wall ambiguity, but in different forms.

This interpretation is consistent with transient-based plane-estimation approaches in the literature. For example, Jungerman et al. [26] estimate planar scene parameters from transient histograms, including the plane orientation through its normal. Our switch-line criterion uses the same geometric quantity, relating the target normal to its intersection with the sampled relay wall and thus identifying when finite-aperture measurements support stable plane-normal estimation.

Although the analytical derivation is written for a single finite planar patch, the same interpretation can be applied locally to more complex scenes. A macroscopic object with moderate curvature can be approximated as a collection of small surface patches, each with its own local tangent plane and local normal. The finite-aperture criterion then predicts which surface portions are expected to be reconstructed more reliably: regions whose local tangent planes satisfy the visibility condition contribute stronger and more stable features, while regions with unfavorable local normals lose measurable information.

**Figure 7:**
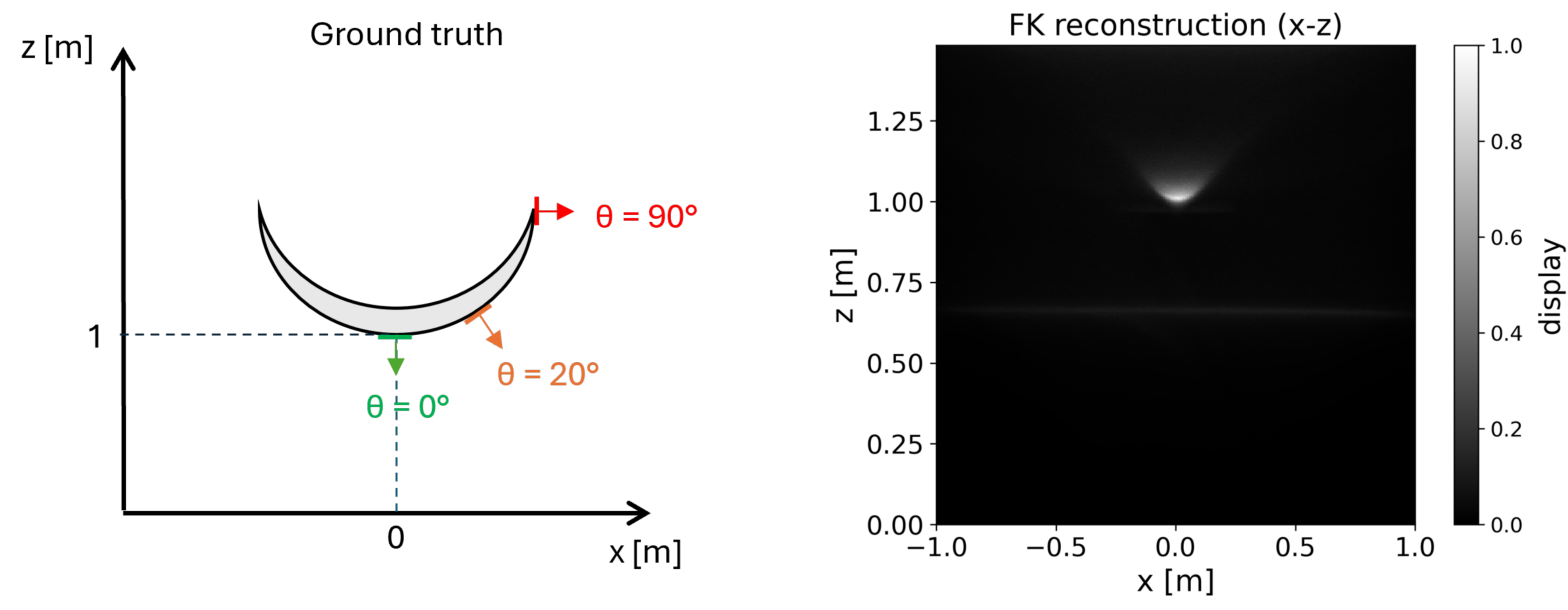
Experimental NLOS reconstruction of a non-planar spherical target. (**Left**): schematic view of the reconstructable spherical cap of a sphere with radius 20 cm, placed 100 cm from the relay wall, and its local surface-normal angle. (**Right**): top-view f-k reconstruction. For this geometry, the switch-line criterion predicts a local visibility range of about $\pm 20^{\circ }$. The reconstructed signal is concentrated around this angular range and becomes much weaker for less favorable local surface orientations.

This local interpretation is illustrated in Figure 7 with an experimental NLOS reconstruction of a spherical target. The sphere contains a continuous range of surface normals. For the wall size and target distance used in this experiment, the switch-line criterion predicts an angular visibility range of about $\pm 20^{\circ }$ for the local tangent planes. Consistently, by inspecting the reconstructed intensity on a central slice of the sphere, we observe that the recovered signal is concentrated within approximately the same angular range and rapidly decreases for larger tangent angles. This behaviour supports the local interpretation of the planar criterion and suggests how it can guide the analysis of moderately curved or composite objects. The main experimental parameters are reported in Table 4.

The confocal setting used here was chosen because it allows a closed-form transition model and gives a clear interpretation of the finite-aperture visibility limit. It is also experimentally relevant, since confocal NLOS acquisition with single-pixel SPAD detection is widely used in laboratory systems. Extending the closed-form analysis to non-confocal geometries is an important direction for future work.

The proposed criteria also provide practical design guidance. The sampled wall region should be positioned so that the most informative transient features remain inside the aperture as the target rotates. This informative region depends on the target shape, its distance from the wall, and its lateral position. Ideally, the sampled wall region should follow the motion of this region as the target rotates. When this is not possible, the nonzero Fisher information regions indicate where yaw information can still be recovered, although at the cost of a larger detected mean photon number or a longer integration time. Once the informative wall region is sufficiently sampled, increasing the wall sampling density mainly improves the lateral resolution of the reconstructed target, rather than changing the angular feature visibility.

The main value of the presented results is the identification of geometric and information-based limits that belong to the measurement itself. The present analysis is thus the first step toward a broader description of the conditions for stable NLOS recovery, indicating when recovery becomes ambiguous and how this transition depends on wall aperture and scene geometry.

Future work will address systematic experimental validation of the yaw threshold criterion with controlled rotations, the extension to non-confocal acquisition geometries, the analysis of more general non-planar scenes through local surface patch approximations, and the inclusion of additional detector and calibration non-idealities. 

## Figures and Tables

**Table 1:** Experimental and numerical parameters for confocal reconstruction tests.

Category	Quantity	Value
Target geometry	Target type	USAF-style paper chart
	Target yaw	$22^{\circ },53^{\circ }$
	Target size [ℓ×h]	$80\times 56\;{\mathrm{cm}}^{2}$
	Target distance	100 cm
	Horizontal anchor	100 cm
Relay wall	Wall area	$200\times 200\;{\mathrm{cm}}^{2}$
	Sampling grid	128×128
	Spatial step	Δx=Δz≃1.56 cm
	Spatial extent	x,z=0–200 cm
Time grid	Number of temporal samples	300
	Temporal bin width	50 ps
	Temporal window	t=0–14.95 ns

**Table 2:** Main experimental components used for the confocal validation measurements.

Component	Manufacturer and Location
Pulsed fiber laser, KATANA, 532 nm	NKT Photonics A/S, Birkerød, Denmark
Single-pixel SPAD detector, PDM Series	Micro Photon Devices, Bolzano, Italy
Fiber collimator, C40FC-A	Thorlabs Inc., Newton, NJ, USA
Two-axis galvo scanning system	Thorlabs Inc., Newton, NJ, USA
Scanner-control electronics	National Instruments Corp., Austin, TX, USA
TCSPC module, Time Tagger	Swabian Instruments GmbH, Stuttgart, Germany

**Table 3:** Summary of the numerical parameters and photon statistics and quantities used for the Fisher information analysis.

Category	Quantity	Value
Target	Target type	isotropic planar patch
	Target distance from wall	100 cm
	Target size [ℓ×h]	$30.0\times 40.0\;{\mathrm{cm}}^{2}$
Relay wall	Wall area	$60\times 60\;{\mathrm{cm}}^{2}$
	Sampling grid	60×60
	Spatial step	Δx=Δy≃1.017 cm
Time sampling and response	Temporal window	5.617–8.679 ns
	Temporal samples	$n_{t}=1227$
	Temporal bin width	2.50 ps
	Gaussian broadening	$\sigma_{t}=10\;\mathrm{ps}$
Yaw sampling	Angular range	[−π/2,π/2]
	Number of angular samples	401
	Angular step	$\Delta \theta ≃0.45^{\circ }$
Transient parameters	Background per wall-time bin	$\beta =1.0\times 10^{-6}$
	Integrated background	$B_{\mathrm{tot}}=4.4172$
	Mean integrated signal	$\langle S_{\mathrm{tot}}\rangle =3.87\times 10^{-2}$
	Mean integrated SNR	$1.83\times 10^{-2}$

**Table 4:** Experimental parameters used for the spherical-target NLOS reconstruction.

Category	Quantity	Value
Target	Target type	Diffusive sphere
	Target radius	20 cm
	Target distance from relay wall	100 cm
Relay wall	Wall area	$200\times 200\;{\mathrm{cm}}^{2}$
	Sampling grid	512×512
	Spatial step	Δx≃Δz≃0.39 cm
Acquisition	Temporal bin width	10 ps
	Temporal samples	1000
	Integration time per point	0.15 s
	Laser repetition rate	10 MHz

## Data Availability

The original contributions presented in this study are included in the article and Supplementary Material. Further inquiries can be directed to the corresponding author.

## Supplementary Materials

The following supporting information can be downloaded at: https://www.mdpi.com/article/10.3390/jimaging12060248/s1, Figure S1: Fixed wall-bin geometry and representative block transient at $\theta =0^{\circ }$; Figure S2: Full transient at the same wall bin for $\theta =0^{\circ }$; Figure S3: Fixed wall-bin geometry and full transient at $\theta =14.5^{\circ }$; Figure S4: Fixed wall-bin geometry and full transient at $\theta =45^{\circ }$; Figure S5: Fixed wall-bin geometry and full transient at $\theta =75^{\circ }$; Figure S6: Reduced symmetry about the same-height switch line for mirrored wall bins.

## Abbreviations

The following abbreviations are used in this manuscript:

NLOS	Non-line-of-sight
FI	Fisher Information
