# Supplementary material for: Finite-Aperture Limits for Yaw Estimation in Confocal Non-Line-of-Sight Imaging
Source: J Imaging. 2026 Jun 2;12(6):248. doi: 10.3390/jimaging12060248 (PMC13300989; doi:10.3390/jimaging12060248)
Supplement: Supplementary file 1 [file jimaging-12-00248-s001.zip › jimaging-4282053-Supplementary materials-2.0 .pdf]

Supplementary Materials
Closed Form Confocal Transient Model, Geometric Interpretation, and Fisher Information for Yaw Estimation

1 Scope and notation

This supplementary document presents the closed form forward model, and the geometric interpretation of the transient volume used to study yaw estimation for a finite planar patch in confocal non line of sight imaging.

The relay wall lies in the plane $y = 0$. The hidden patch lies in the half space $y > 0$. It has width ℓ, height h, stand off distance $d > 0$, lateral position x_0, vertical position z_0, and yaw angle θ about the $\hat{\mathbf{z}}$ axis. In the confocal setting, each sampled wall bin acts as both source and detector. A wall bin is denoted by

$$\mathbf{w} = (x_w, 0, z_w). \tag{1}$$

The corresponding offsets of the patch relative to that wall bin are

$$x_o = x_0 - x_w, \qquad z_o = z_0 - z_w. \tag{2}$$

Throughout the document, c is the speed of light and $v_{\text{eff}} = c/2$ is the effective speed in the confocal exploding reflector convention.

Two wall features will be used repeatedly. The *switch line* is the wall line where the ordering of two same height endpoint contributions changes. The *interior foot region* is the wall subset whose orthogonal projection onto the infinite plane falls inside the finite patch.

2 Closed form forward model

2.1 Fronto parallel half facet kernel

We first consider a fronto parallel half facet with local coordinates

$$(x, z) \in [0, L] \times [0, H], \tag{3}$$

placed at distance $\lambda > 0$ from the wall. In the confocal geometry, equal delay curves on the plane are circles centered at the orthogonal foot of the wall point, with radius

$$r(t) = \sqrt{t^2 - \lambda^2}. \tag{4}$$

Assuming a unit pulse and the usual inverse distance weighting, the continuous transient is

$$f(t; L, H, \lambda) = \int_0^L dx \int_0^H dz \, \frac{\delta\big(\sqrt{x^2 + z^2 + \lambda^2} - t\big)}{(x^2 + z^2 + \lambda^2)^2}. \tag{5}$$

Evaluating the integral in polar coordinates gives

$$f(t; L, H, \lambda) = \frac{1}{t^3} \left[\arcsin\left(\min\left\{ 1, \frac{H}{\sqrt{t^2 - \lambda^2}} \right\} \right) - \arccos\left(\min\left\{ 1, \frac{L}{\sqrt{t^2 - \lambda^2}} \right\} \right) \right] \times \mathbf{1}_{[\lambda,\, \sqrt{\lambda^2 + L^2 + H^2}]}(t). \tag{6}$$

This kernel is the building block used below to write the exact transient of a finite yawed patch.

2.2 Yawed finite patch and orthogonal foot

The finite patch is parameterized as

$$\mathbf{r}(u,v) = (x_o, d, z_o) + u(\cos\theta, \sin\theta, 0) + v(0,0,1), \qquad u \in \Big[-\frac{\ell}{2}, \frac{\ell}{2}\Big], \quad v \in [0,h]. \tag{7}$$

Its outward unit normal is

$$\mathbf{n}(\theta) = (-\sin\theta, \cos\theta, 0). \tag{8}$$

The orthogonal distance from the wall origin to the infinite extension of the plane is

$$\lambda(\theta, x_o, d) = d\cos\theta - x_o \sin\theta. \tag{9}$$

The orthogonal foot on the infinite plane has in plane coordinates

$$u_\infty = -(x_o\cos\theta + d\sin\theta), \qquad v_\infty = -z_o. \tag{10}$$

If (u_∞, v_∞) lies inside the finite support, then the first contributing point is an interior point of the patch. Otherwise it is the clamped point

$$u_P = \mathrm{clip}\Big(u_\infty, \Big[-\frac{\ell}{2}, \frac{\ell}{2}\Big]\Big), \qquad v_P = \mathrm{clip}(v_\infty, [0,h]). \tag{11}$$

Its 3D position is

$$\mathbf{P} = (x_o, d, z_o) + u_P(\cos\theta, \sin\theta, 0) + v_P(0,0,1). \tag{12}$$

2.3 Exact block assembly

The finite patch is split at (u_P, v_P) into four rectangles. The equal delay circles, however, remain centered at (u_∞, v_∞) and not at the clamped point. This is why the exact transient cannot be written only in terms of block widths and heights when the orthogonal foot leaves the finite support.

For any first quadrant rectangle $[a_0, a_1] \times [b_0, b_1]$ with $0 \le a_0 \le a_1$ and $0 \le b_0 \le b_1$, define

$$\begin{aligned} f\big(t; [a_0,a_1],[b_0,b_1],\lambda\big) = {} & f(t; a_1, b_1, \lambda) - f(t; a_0, b_1, \lambda) \\ & - f(t; a_1, b_0, \lambda) + f(t; a_0, b_0, \lambda), \end{aligned} \tag{13}$$

where the terms on the right are given by equation (6). Intervals on the negative semiaxis are reflected by symmetry. Intervals crossing the origin are split at zero and the resulting non empty pieces are summed.

Let

$$u_L = -\frac{\ell}{2}, \qquad u_R = \frac{\ell}{2}, \qquad v_B = 0, \qquad v_T = h. \tag{14}$$

The four block intervals, written relative to the orthogonal foot on the infinite plane, are

$$\mathcal{U}_1 = [u_P - u_\infty,\, u_R - u_\infty], \qquad \mathcal{U}_2 = [u_L - u_\infty,\, u_P - u_\infty], \tag{15}$$

$$\mathcal{V}_1 = [v_P - v_\infty,\, v_T - v_\infty], \qquad \mathcal{V}_2 = [v_B - v_\infty,\, v_P - v_\infty]. \tag{16}$$

The exact transient generated by one confocal wall bin is therefore

$$\tau(t; \theta, \ell, h, d, x_o, z_o) = \sum_{i=1}^{2}\sum_{j=1}^{2} f\big(t; \mathcal{U}_i, \mathcal{V}_j, \lambda(\theta, x_o, d)\big). \tag{17}$$

When the orthogonal foot is interior to the patch, one has $u_P = u_\infty$ and $v_P = v_\infty$, and the usual width and height assembly is recovered as a special case.

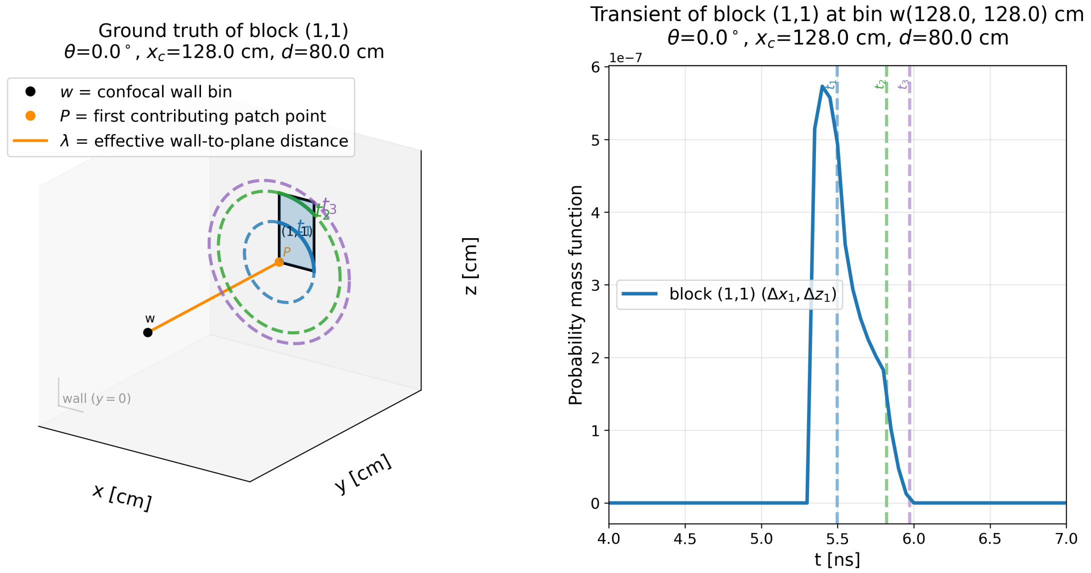

Figure S1: Fixed wall bin geometry at $\theta = 0°$. Left: patch plane geometry for one confocal wall bin, showing the orthogonal foot P_∞, the first contributing point P, and the block partition. Right: transient of one representative block. The onset is set by the first contact of the equal delay circle with the block, while later contacts with an edge or a corner produce the visible slope changes.

2.4 Transient volume on the wall

Sampling the relay wall on a two dimensional confocal grid gives the transient volume

$$V(t, z_w, x_w; \theta) = \big(\tau(\,\cdot\,; \theta, \ell, h, d, x_0 - x_w, z_0 - z_w) * h\big)(t), \tag{18}$$

where $h(t)$ is the detector temporal impulse response function and $*$ denotes convolution in time.

If $V[n, m, k]$ denotes the temporally binned transient volume on a uniform wall grid, the globally normalized transient defines the probability mass function

$$\widetilde{V}_{n,m,k}(\theta) = \frac{V_{n,m,k}(\theta)}{\sum_{n,m,k} V_{n,m,k}(\theta)}. \tag{19}$$

For a uniform wall grid, the wall cell area $\Delta A_w = \Delta x_w \Delta z_w$ cancels exactly in this normalization. It must be retained only when approximating unnormalized wall integrals, or when the wall sampling is non uniform.

2.5 Representative single wall bin examples

The forward model is easier to read by looking at one wall bin and following how its transient changes with yaw. At a fixed wall bin, the waveform is determined by three quantities: the distance λ, the position of the orthogonal foot relative to the finite support, and the resulting block partition of the patch.

As θ increases, the orthogonal foot moves across the patch plane. Once it reaches a lateral boundary, the first contributing point is clamped to that boundary and one or more blocks collapse.

For larger yaw, the model enters a side clamped regime. The same side boundary remains active over a finite angular range, so the transient keeps changing but its structure changes more smoothly.

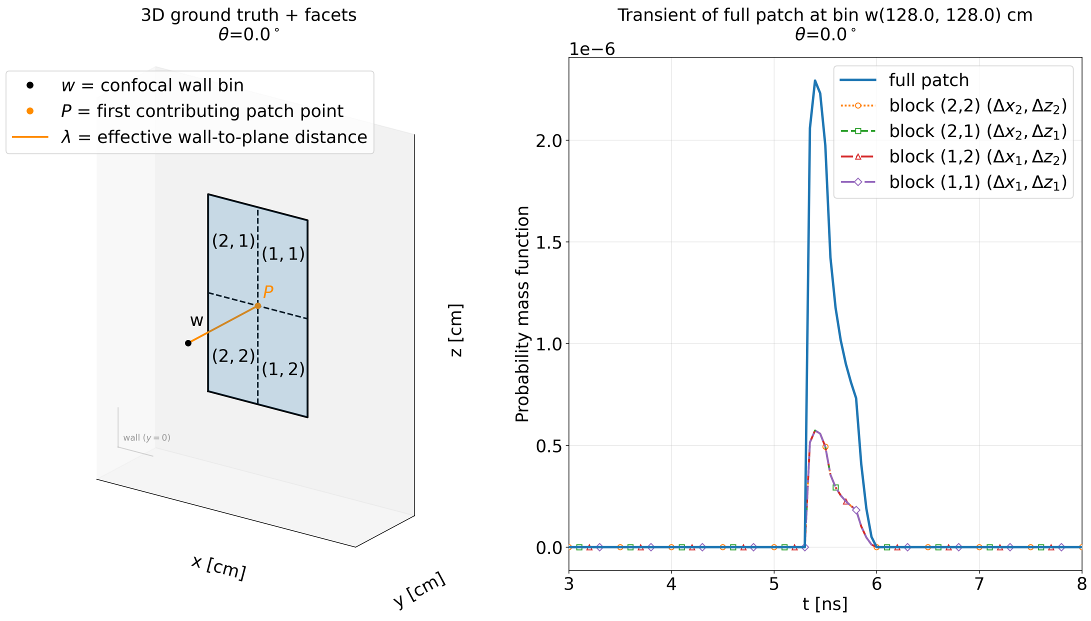

Figure S2: Full transient at the same wall bin for $\theta = 0°$. The total response is the sum of the four block contributions in equation (17). In this symmetric case, the upper pair overlap and the lower pair overlap, so only two distinct waveforms remain in the total transient.

Taken together, figures S1 to S5 give the local picture behind the full transient volume. The complete dataset is obtained by evaluating the same single bin mechanism over all wall positions, before adding a smoothing term.

2.6 Interior foot region and one sided gate

The interior foot region is the wall subset for which the orthogonal projection onto the infinite plane falls inside the finite patch. In the present notation, it is the set of wall bins satisfying

$$u_\infty(x_w, \theta) \in \left[-\frac{\ell}{2}, \frac{\ell}{2}\right], \qquad v_\infty(z_w) \in [0, h]. \tag{20}$$

Inside this region the first contributing point is interior. Outside it, the first contributing point is clamped to an edge or a corner.

3 Geometric interpretation of the transient volume

3.1 A single transient as a weighted radial profile

For a fixed wall bin $W \equiv (x_w, z_w)$, the transient

$$t \mapsto \tau(t; \theta, \ell, h, d, x_0 - x_w, z_0 - z_w) \tag{21}$$

is determined by the finite patch support sampled by equal delay circles around the orthogonal foot on the infinite plane. Its first and last possible returns are

$$t_{\min}(W) = \frac{\|\mathbf{w} - \mathbf{P}\|}{v_{\text{eff}}}, \qquad t_{\max}(W) = \frac{1}{v_{\text{eff}}} \max_{\mathbf{r} \in \Pi_\theta^{\text{fin}}} \|\mathbf{w} - \mathbf{r}\|, \tag{22}$$

where Π_θ^{fin} denotes the finite patch.

A single transient is therefore not a unique signature of the hidden patch. It is a weighted radial profile around the orthogonal foot: it contains the onset, the trailing edge, the support width, and the internal support transitions, but not the full two dimensional arrangement of the patch on the plane.

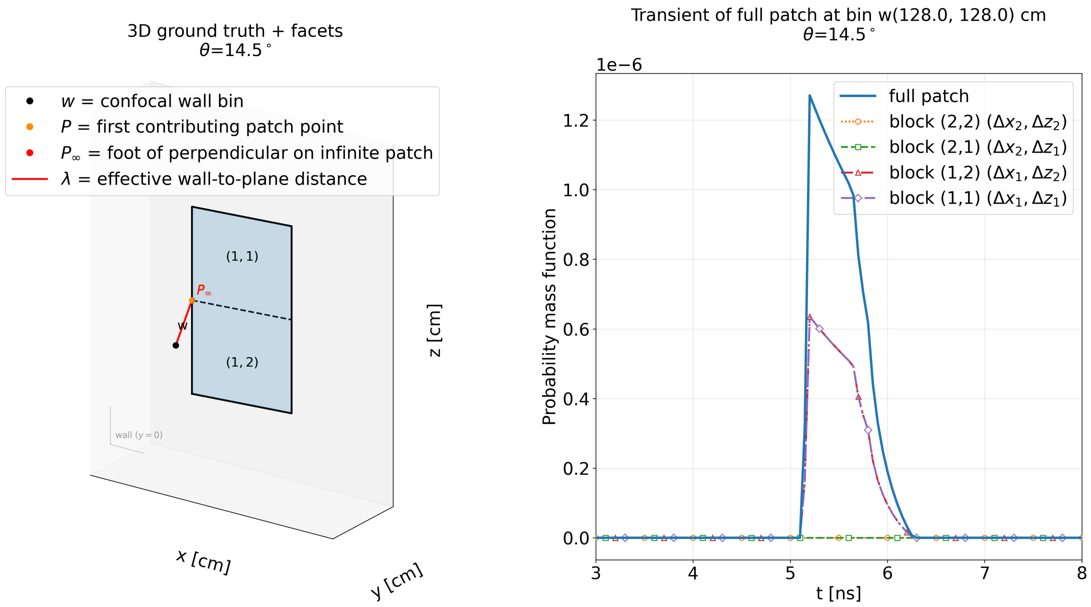

Figure S3: Fixed wall bin geometry at $\theta = 14.5°$. The first contributing point has reached the left boundary of the patch, so the blocks on that side no longer contribute. The transient becomes simpler and its onset shifts accordingly.

3.2 Why the full wall measurement is richer

The full transient volume breaks many single bin ambiguities because each wall bin induces a different local geometry through the offsets in equation (2). The wall measurement is therefore a family of different radial observations of the same patch, not a repetition of one ambiguous histogram.

This also explains why finite aperture clipping matters. As yaw varies, the onset and trailing edge structures move across the wall. If the sampled wall still contains the relevant regions, the data preserve the wall comparisons that constrain orientation. If those regions are clipped, the measured volume becomes a cropped version of the ideal one.

3.3 Switch line and geometric visibility limit

For yaw rotations, the main support change comes from the competition between same height endpoint pairs. The wall location where the two endpoint contributions exchange their order of arrival is the switch line

$$x^\star(\theta) = x_0 + d \tan\theta. \tag{23}$$

It is a vertical line on the wall because the equal range condition is independent of z_w for same height endpoints.

If the sampled wall spans $[x_{w,\min}, x_{w,\max}]$, an internal switch is present only if

$$x_{w,\min} \le x^\star(\theta) \le x_{w,\max}. \tag{24}$$

For $\theta \in [0, \pi/2]$, this gives the yaw window

$$\theta \in \left[\arctan\left(\frac{x_{w,\min} - x_0}{d}\right), \arctan\left(\frac{x_{w,\max} - x_0}{d}\right)\right]. \tag{25}$$

When the switch line lies inside the sampled wall, the data still contain bins on both sides of the endpoint swap. When it leaves the aperture, the wall records only a cropped part of the ideal support, and unique planar reconstruction from support information alone is lost.

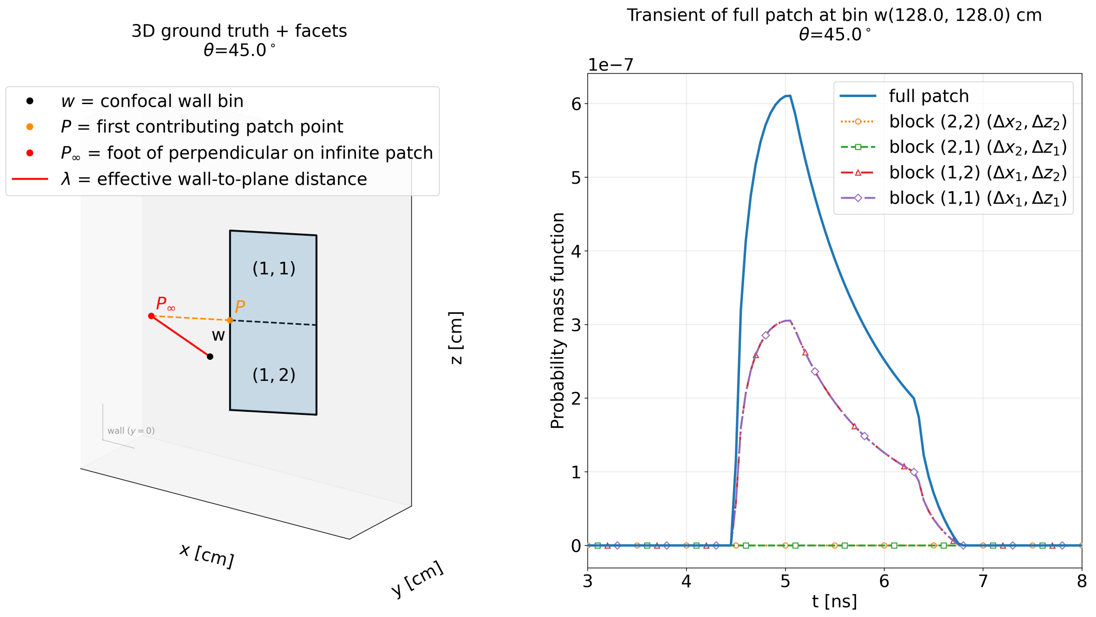

Figure S4: Fixed wall bin geometry at $\theta = 45°$. The first contributing point is clamped to a side edge. In this regime the set of active blocks is already fixed, and the transient changes mainly through the relative weight of the surviving terms.

3.4 Symmetries and reduced symmetry about the switch line

Several useful symmetries remain in the ideal model.

For pure yaw, the transient volume is vertically mirror symmetric about the patch mid height:

$$V\Big(t, z_0 + \frac{h}{2} + \Delta z, x_w; \theta\Big) = V\Big(t, z_0 + \frac{h}{2} - \Delta z, x_w; \theta\Big). \tag{26}$$

For the fronto parallel case $\theta = 0$, the volume is laterally symmetric about the patch center:

$$V(t, z_w, x_0 + \Delta x; 0) = V(t, z_w, x_0 - \Delta x; 0). \tag{27}$$

A yawed patch also admits a paired symmetry between opposite yaw angles:

$$V(t, z_w, x_w; +\theta) = V\big(t, z_w, 2x_0 - x_w; -\theta\big). \tag{28}$$

At fixed nonzero yaw, however, there is generally no exact raw mirror symmetry about the switch line. Wall bins symmetric with respect to $x^\star(\theta)$ have mirrored local support geometry, but different onset distances:

$$\lambda(x^\star + \delta) = d \sec\theta + \delta \sin\theta, \qquad \lambda(x^\star - \delta) = d \sec\theta - \delta \sin\theta. \tag{29}$$

Therefore

$$V(t, z_w, x^\star + \delta; \theta) \neq V(t, z_w, x^\star - \delta; \theta) \qquad \text{in general.} \tag{30}$$

A weaker symmetry appears after removing the wall dependent onset shift. Introducing the local radial coordinate

$$\rho = \sqrt{(v_{\text{eff}} t)^2 - \lambda^2}, \tag{31}$$

and compensating the leading radiometric factor t^3, transient pairs from mirrored wall bins nearly collapse onto the same curve in discrete simulations and coincide in the continuous model.

Figure S6 illustrates this reduced symmetry for a representative pair of wall bins chosen on opposite sides of the switch line. In the raw time domain, the two traces are not mirror copies

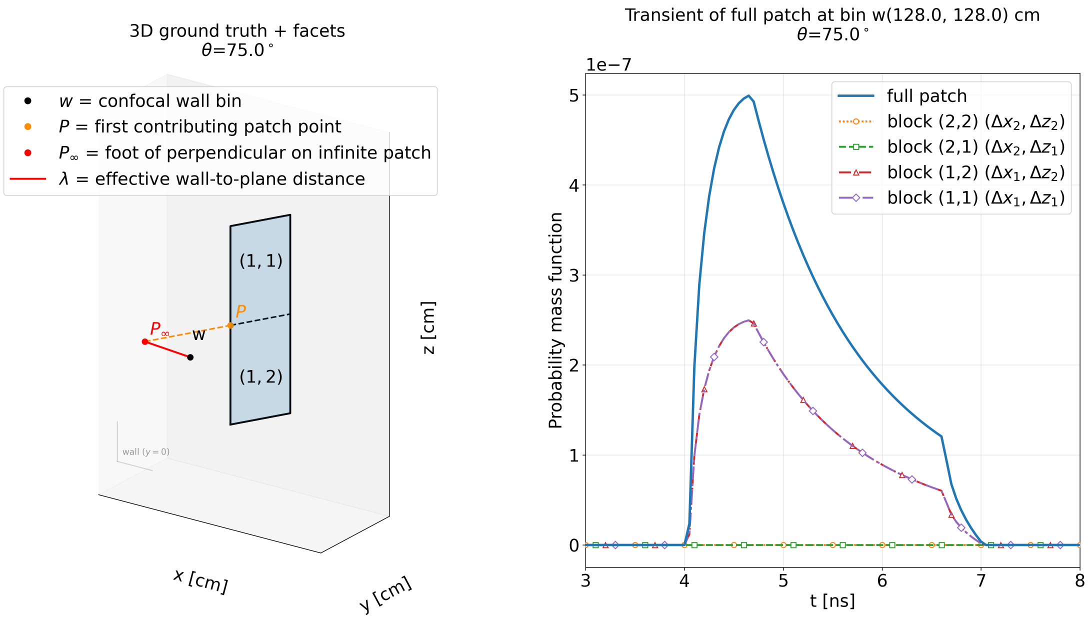

Figure S5: Fixed wall bin geometry at $\theta = 75°$. The first contributing point remains clamped to the same side boundary as in figure S4. The qualitative transient structure is therefore preserved, even though the support intervals and weights still vary with yaw.

because they have different values of λ and therefore different onset times. After reparameterization in ρ, the two curves become much more similar. After the additional t^3 compensation and normalization, they almost overlap. This comparison makes clear that the switch line is not an axis of exact symmetry in the measured transient volume, but rather the natural reference for a reduced symmetry that emerges once the wall dependent onset shift is removed.

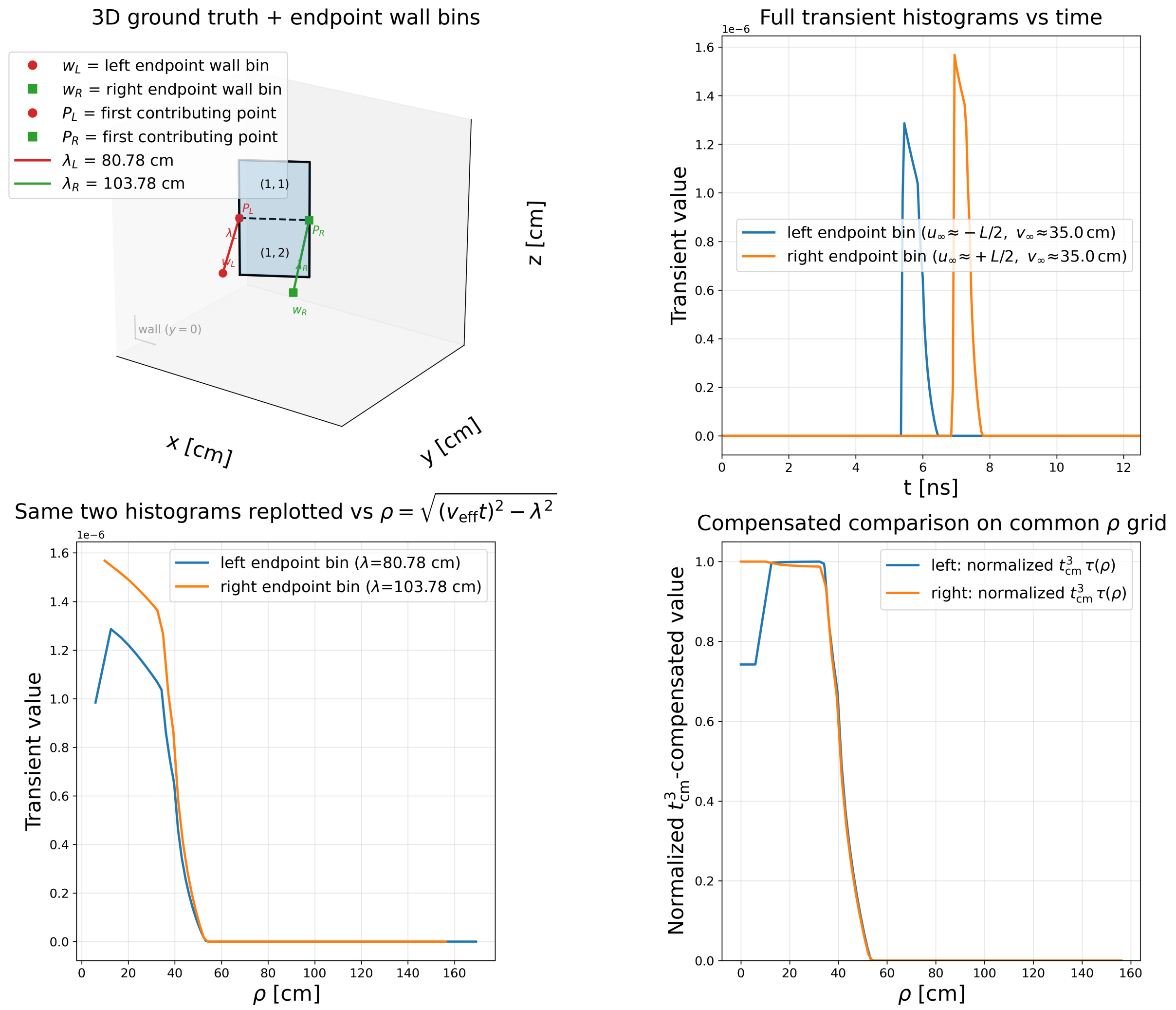

Figure S6: Reduced symmetry about the same height switch line for a representative pair of mirrored wall bins. Top left: geometry of the yawed finite patch and the two selected wall bins. Top right: transient histograms versus absolute time t. Bottom left: the same histograms plotted versus the local radial coordinate $\rho = \sqrt{(v_{\mathrm{eff}}t)^2 - \lambda^2}$. Bottom right: comparison after t^3 compensation and normalization on a common ρ grid. In the continuous model the two compensated curves coincide exactly. The small residual mismatch in the discrete simulations comes from wall sampling, temporal binning, and interpolation.
